# A Severe Case of Arthrofibrosis After Anterior Cruciate Ligament (ACL) Reconstruction in a 25-Year-Old Female Hispanic Patient: A Case Report

**DOI:** 10.7759/cureus.69933

**Published:** 2024-09-22

**Authors:** Edrick Ujaque Rivera, Felix Rivera Troia, Gerardo Perez Roman

**Affiliations:** 1 Internal Medicine, University of Medicine and Health Sciences, Basseterre, KNA; 2 Surgery, University of Medicine and Health Sciences, Basseterre, KNA; 3 Genetics, Ponce Health Sciences University, Ponce, PRI; 4 Sports Medicine, Dr. Gerardo Perez Roman Orthopedics, San Juan, PRI

**Keywords:** acl repair, acl tear, female athlete, hispanic population, knee arthrofibrosis

## Abstract

Arthrofibrosis is the most common postoperative complication of anterior cruciate ligament (ACL) reconstruction. It is caused by an exaggerated immune reaction to a pro-inflammatory trigger that causes abnormal periarticular fibrosis and joint stiffness. The shoulder, elbow, and knee are especially prone to this condition, often following trauma, surgery, or adhesive capsulitis. We describe the case of a 25-year-old Hispanic female who presented to the clinic with knee instability after experiencing a twisting and popping sensation in her left knee while playing tennis. Physical examination revealed increased anterior tibial translation, and imaging confirmed a torn ACL. She subsequently underwent ACL reconstruction using the bone-patellar tendon-bone graft technique. However, during her follow-up appointments, she reported persistent difficulties with active and passive range of motion in both flexion and extension despite undergoing appropriate physical therapy. Now, three months post-operation, she continues to experience limited knee range of motion. To our knowledge, there are only a few reports of arthrofibrosis in this particular age group and demographic undergoing this procedure. Our objective is to contribute this case to the scientific community, aiming to encourage future studies and gather epidemiological data on this topic.

## Introduction

Arthrofibrosis is a condition characterized by the accumulation of scar tissue around a joint, often following a traumatic injury or surgical intervention. The knee is one of the less commonly affected joints. In the United States, only 2% of known cases each year develop as a complication of surgical treatment [1]. Arthrofibrosis occurs in 2%-35% of cases, with a higher prevalence in female patients compared to males. Frozen shoulder is the most commonly affected condition, accounting for the majority of cases. Among these, 10% result from surgical interventions, while 90% are associated with non-surgical management [2].

After trauma or surgical manipulation of a joint, the wound-healing process typically begins with the activation of the immune system to repair the damage. The process starts with the formation of a provisional fibrin clot, followed by the arrival of immune cells such as platelets, neutrophils, CD4 helper T cells, and macrophages at the injury site. These cells, guided by immunomodulators like IL-6, IL-1, transforming growth factor β1 (TGF-β1), and fibroblast growth factor (FGF), initiate the immune response. Tissue remodeling then occurs through fibroblast migration and the synthesis of extracellular matrix (ECM) proteins. While this process is usually well-organized, dysregulation can occur due to altered cytokine levels, leading to the pathological formation of disorganized ECM proteins, a condition known as fibrosis [3].

A study investigating risk factors for arthrofibrosis following ACL reconstruction found a tendency for the condition to occur more frequently in female patients who underwent surgical repair within a month of injury, particularly when the bone-patellar-tendon-bone technique was used [4]. Potential risk factors identified included an intensive rehabilitation process, prolonged immobilization, and concomitant meniscus tears. Among the patients studied, 89% of those affected were 18 years old or older, with 63% being male and 37% female. Within the athletic group analyzed, soccer players exhibited the highest incidence of arthrofibrosis [5,6].

## Case presentation

**Figure 1:**
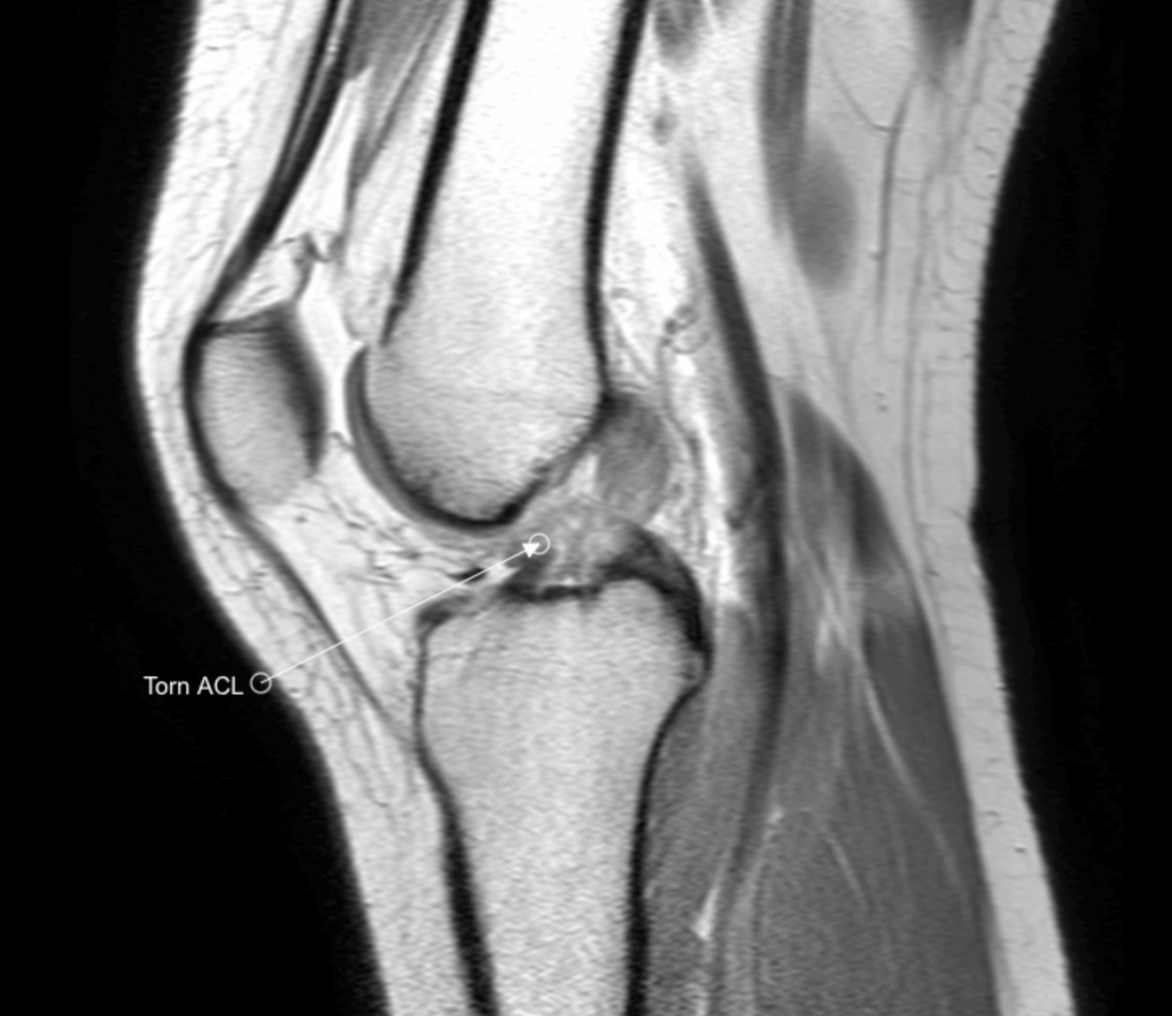
PD MRI sagittal view of the left knee showing torn ACL PD: Proton density; ACL: Anterior cruciate ligament.

**Figure 2:**
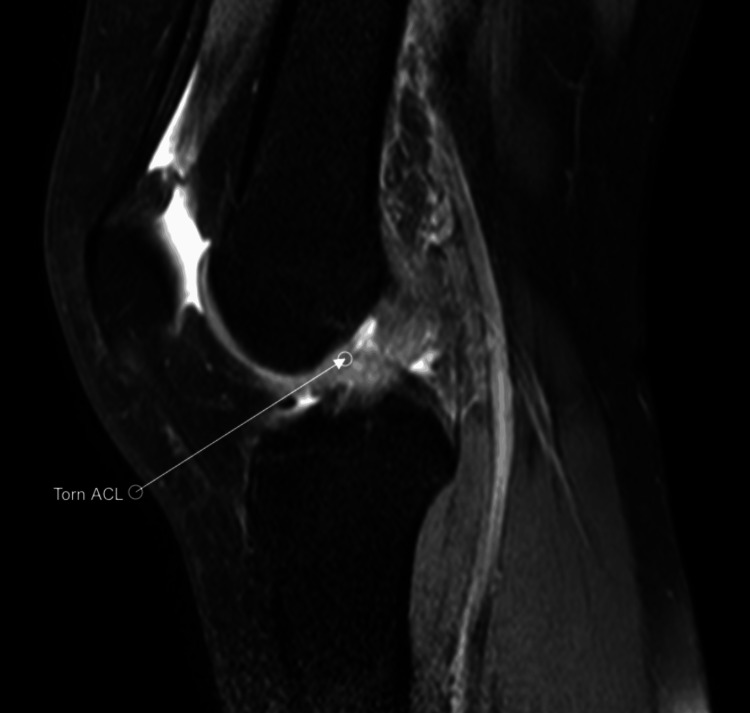
T2 MRI sagittal view of the left knee showing torn ACL ACL: Anterior cruciate ligament.

A 25-year-old female arrived at the clinic complaining of left knee instability after a twisting and popping sensation while playing tennis. Her past medical history is significant for papillary thyroid cancer. Her only past surgical history is a thyroidectomy. She currently takes levothyroxine for thyroid hormone supplementation and anti-inflammatory medication for her knee pain. Physical exam was significant for a positive anterior drawer sign and a positive Lachman’s test as well as knee instability. Magnetic resonance imaging (MRI) was ordered, and the impression showed a completely torn ACL along its mid to distal third portion (Figures 1, 2).

The patient was scheduled for surgery using the bone-patellar tendon-bone graft technique. The surgery went well with no intraoperative complications. In her first follow-up appointment, six days post-surgery, the patient began complaining of knee tightness in both flexion and extension. She was advised to begin physical therapy (PT) and continue with her scheduled follow-up appointments. The patient continued to complain of knee tightness despite adequate PT and was scheduled for knee manipulation under anesthesia to further assess the restricted range of motion (ROM). During manipulation, crackling noises were heard from the knee, and tightness was felt during passive ROM, indicating that the patient had developed severe arthrofibrosis.

Initially, she was scheduled for PT three times a week but did not respond adequately. She has since been transitioned to a PT regimen twice daily, five days a week. She continues to make progress slowly, improving both active and passive ROM, and is currently tolerating the treatment well.

## Discussion

A retrospective study on arthrofibrosis identified a history of high-level competitive sports as the sole risk factor for developing the condition. Conversely, protective factors include being younger than 18 years and receiving postoperative inpatient management [5]. Among the surgical procedures known to be complicated by arthrofibrosis, frozen shoulder emerges as the most frequently encountered condition. In contrast, elbow trauma is the least likely to result in the development of arthrofibrosis, making it the least affected joint [2]. The median age of patients undergoing revision total knee arthroplasty (TKA) for arthrofibrosis is 64.7 years, indicating that arthrofibrosis commonly affects older adults following knee surgery [7].

Anterior cruciate ligament (ACL) injuries are one of the most common and severe knee injuries across sports [8]. One epidemiologic study followed ACL injuries among high school athletes based on sports and sex. The study results showed an injury rate of 6.5 per 100,000 athlete exposures. They found that the injury rate was higher during competition when compared to practice with a relative risk (RR) of 7.3% and confidence interval (CI) of 6.08, 8.68 and that girls had a higher rate of injury when compared to boys with RR of 3.4% and CI of 2.64, 4.47. The sport where most of these injuries occurred was soccer, followed by basketball [9].

To better understand ACL injuries and reconstruction, a brief review of the anatomy of the ligament is essential. The ACL is one of two cruciate ligaments that provide knee stabilization in the sagittal plane. It originates from the tibial plateau, between and anterior to the intercondylar eminences, and inserts in the posteromedial aspect of the lateral femoral condyle [10]. The ACL is often described as a double-bundle structure due to the way it divides the tissue into multiple bundles. Traditionally, these two bundles are identified as the anteromedial and posterolateral bundles. They are distinguished by a tissue sheath separating them and their distinct insertion sites. Functionally, the anteromedial bundle of the ACL is more active during deep knee flexion, while the posterolateral bundle is mostly functional near full extension. Furthermore, the anteromedial bundle provides more support under anterior tibial forces, whereas the posterolateral bundle is more involved in resisting rotational forces [11].

ACL reconstruction is accomplished using various techniques such as quadricep tendon autografts, hamstring tendon autografts, and patellar tendon autografts. Of these, the two most commonly employed techniques are hamstring and patellar tendon autografts. A meta-analysis performed in 2017 by Samuelsen et al. compared the outcomes of each technique in 47,613 patients. Their results showed that 2.80% of patients who underwent patellar tendon autografts experienced rupture when compared to 2.84% in the hamstring group (odds ratio = 0.83; 95% CI = 0.72-0.96; p = 0.01). Albeit low failure rates for both techniques, they concluded that hamstring autografts failed at a higher rate than patellar tendon autografts [12]. Another meta-analysis by Migliorini et al. in 2023 compared bone-patellar tendon-bone (BPTB) autografts versus two- and four-strand hamstring tendon autografts for ACL reconstruction in young adults. Their results showed that BPTB autografts led to reduced joint laxity and improved ROM when compared to two- and four-strand hamstring autografts. Moreover, they also reported that four-strand hamstring autografts demonstrated the quickest return to sport, followed by BPTB and two-strand hamstring allografts [13].

Arthrofibrosis is a common complication of ACL reconstruction, with an incidence rate of 2%-35% [1]. The literature provides limited information on the occurrence of arthrofibrosis in young Hispanic women specifically. Some studies have identified risk factors for arthrofibrosis, including female sex, the timing of reconstruction, and the type of graft used [14]. However, nothing specifically mentioned age or Hispanic origin as being risk factors. A case report published in 2023 showed a 45-year-old female patient who presented with knee stiffness after ACL reconstruction. The patient was scheduled for arthroscopic lysis of adhesion and manipulation under anesthesia. They reported improvement in both flexion and extension after the procedure with the help of an intensive physiotherapy regiment and good patient compliance [1]. Nevertheless, this report did not address the patient’s ethnic background.

## Conclusions

Arthrofibrosis remains a challenging complication following ACL reconstruction, with incidence rates varying widely and a noted tendency toward higher occurrences in female patients. This case report highlights a 25-year-old Hispanic female who experienced severe arthrofibrosis despite appropriate surgical intervention and rehabilitation. Her ongoing difficulties with knee ROM underscore the need for increased awareness and tailored management strategies in this demographic. Given the limited literature on arthrofibrosis in young Hispanic women, this case contributes valuable insights into the field, suggesting that further research is necessary to explore potential demographic specific risk factors. Exploring these factors could improve prevention and treatment approaches, ultimately leading to better outcomes for similar patients. This case emphasizes the importance of continued investigation into the epidemiology and management of arthrofibrosis to better address and mitigate this complication in diverse populations.
